# High efficiency dielectric photonic crystal fiber metalens

**DOI:** 10.1038/s41598-020-77821-5

**Published:** 2020-12-01

**Authors:** Myunghwan Kim, Soeun Kim

**Affiliations:** Integrated Optics Laboratory, Advanced Photonics Research Institute, GIST, Gwangju, 61005 Republic of Korea

**Keywords:** Fibre optics and optical communications, Metamaterials

## Abstract

Optical fibers have been utilized in various fields owing to their superior guiding performance. However, the modification of optical properties and light manipulation in fibers are restricted by the limitation of the core and cladding materials. In addition, the spot size of the light is constrained by the diffraction limit. In this study, we propose an all-dielectric metalens patterned on the facet of a photonic crystal fiber. The metasurface, which contains Si pillars, satisfies the required phase diagram for focusing light with high transmission. The proposed metalens has a focal length of 30 µm and achieves an outstanding efficiency of up to 88% at a wavelength of 1.55 µm, which is approximately 5 times higher than that of a metal-based metalens. We believe that this scheme may pave the way for in-fiber metasurface applications.

## Introduction

Optical fibers have been widely used in various light-transmission applications due to their excellent performance in guiding light with negligible loss^1,2^. Despite this superior guiding performance, their functionality is restricted because in-fiber light manipulation and optical property modulation are challenging due to the unchangeable dielectric properties of the core and cladding materials. In addition, the transmitted mode size in fibers is quite large, and attempts to reduce this are hindered by the diffraction limit. Therefore, it is difficult to couple light from fibers to integrated devices, which requires additional components to satisfy mode-matching condition^3^. To overcome these problems, several attempts have been made to extend the functionality of optical fibers. For example, nanostructured elements have been arranged on the fiber facets to tailor the mode properties, and these have been employed in various applications, such as optical plasmonic sensors^4–6^, filters^7^, amplifiers^8^, and, optical tweezers^9^. In particular, optical lenses using plasmonic nanostructures have been actively investigated^10–13^. However, the schemes using annular slits have a limited range of potential applications due to their short focal length and high-order diffraction.

Metasurfaces composed of metal or dielectric aperiodic arrays have attracted specific attention due to their unique properties^14,15^. The different phase delays induced by each meta-atom enable beam steering^16^, focusing^17^, and manipulation^18^. In recent years, optical components composed of integrated in-fiber metasurfaces have been reported, such as in-fiber modulators^19^, linear polarizers^20^, and beam elements^21^. In particular, a metal-based in-fiber metalens composed of a nanostructured aperiodic metal array on a core area, has been experimentally demonstrated for the focusing of circular polarized light^22^. Although this metalens is able to focus light with enhanced optical intensity, its efficiency is only 16.4% due to metal loss. To overcome this problem, we propose an all-dielectric metalens depositing an aperiodic Si pillar array on a large-mode-area photonic crystal fiber (LMA-PCF) with a large core area. Si pillars with different diameters provide the phase change required for the focusing of light with a high transmission efficiency. Because Si loss is negligible at optical communication wavelengths, the proposed metalens can greatly reduce the material loss and achieves an exceptional efficiency of 88% with a numerical aperture (NA) of 0.398. We believe that the proposed all-dielectric in-fiber metasurface scheme can be employed in practical in-fiber applications.

## Results

### Design of the metalens

**Figure 1:**
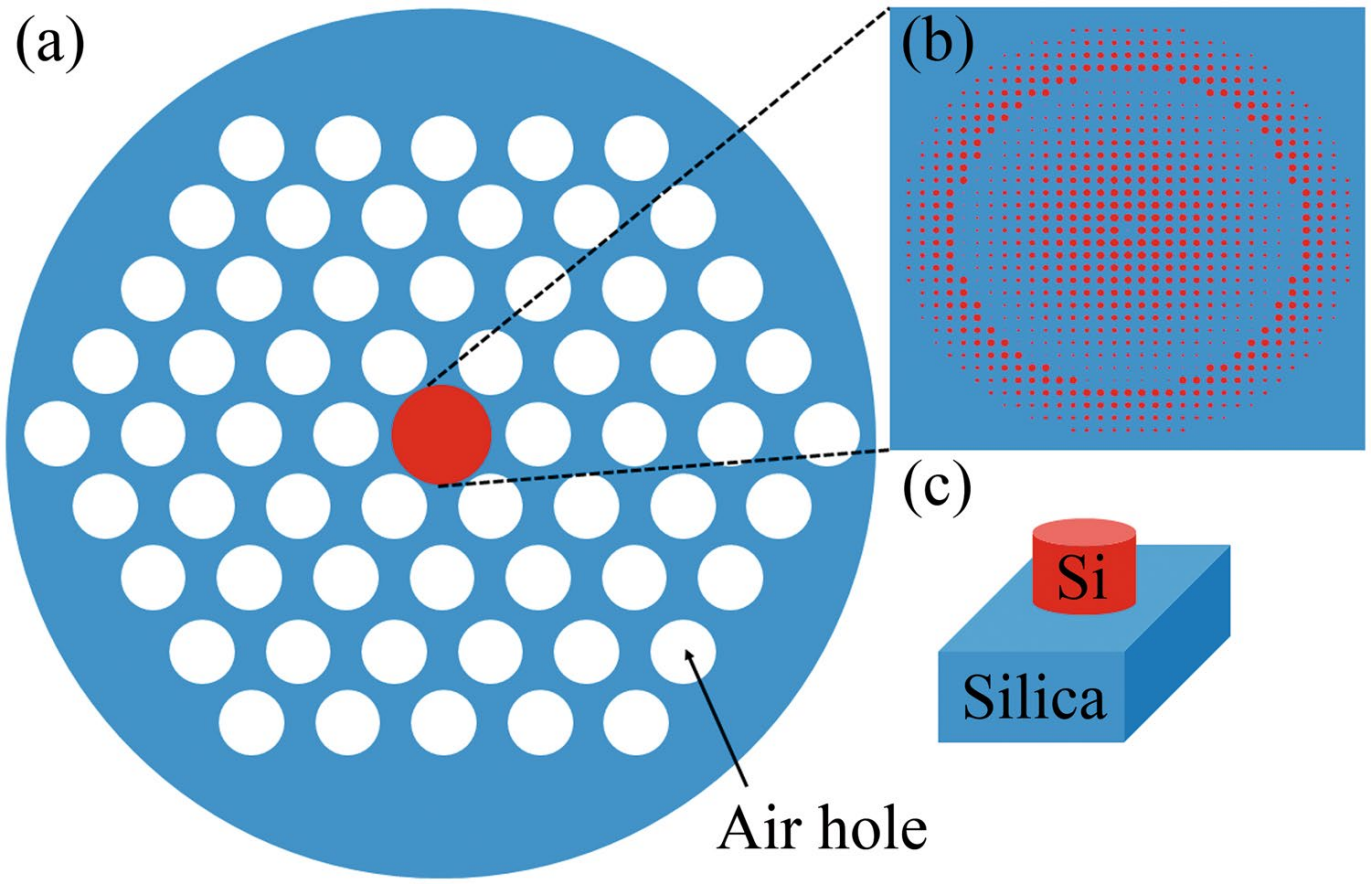
All-dielectric in-fiber metalens. (**a**) 2D schematic diagram of the metalens on a LMA-PCF. (**b**) Elements of the metalens. (**c**) Unit element of the metalens.

We designed the metalens on a LMA-PCF with a large core area while maintaining a single-mode condition. The LMA-PCF consists of a silica core with a large area of 26 µm and hexagonal air holes with a period and diameter of the air hole are 16.4 µm and 4 µm, respectively. It is worth noting that a larger core area is preferred because more meta-atoms could be placed in the core region. Figure 1a,b depict the cross-section of the proposed in-fiber metalens. We consider the Si pillars to be the building blocks of the metalens (Fig. 1c). The refractive indices of Si and silica are assumed to be 3.45 and 1.45, respectively, and the operating wavelength is assumed to be 1.55 µm. This structure allows 877 meta-atoms to be placed in the core region (the distance between meta-atoms is 780 nm) to produce the required phase diagram, thus creating a denser phase profile than a single-mode fiber-based metalens.

**Figure 2:**
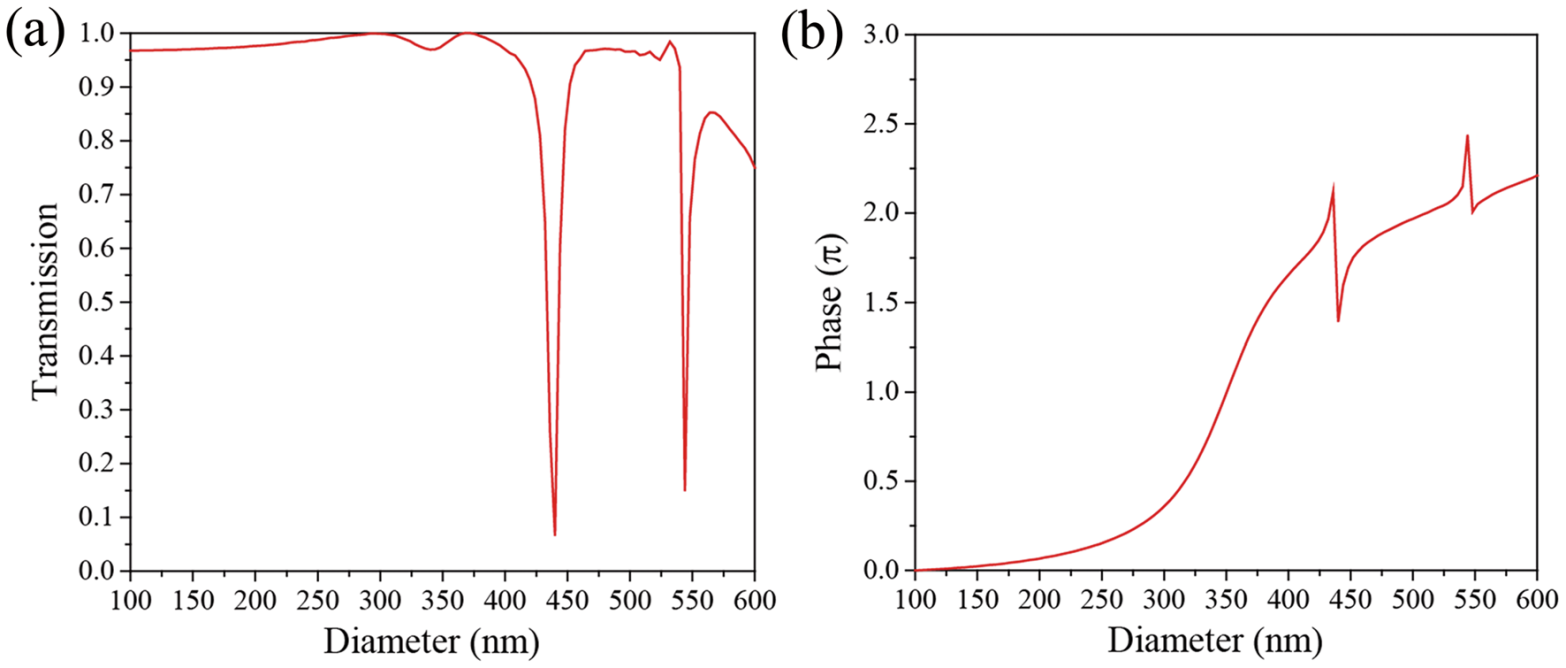
Characteristics of the metasurface. (**a**) Transmission and (**b**) phase delay as a function of the diameter of the periodic Si pillars.

To focus light at a specific focal length, the following phase distribution needs to be obtained^23^:

1 $$\varphi \left( {r,\lambda } \right) = - \frac{2\pi }{\lambda }\left( {\sqrt {r^{2} + f^{2} } - f} \right)$$

where *f* is the focal length, *λ* is the operating wavelength, and *r* is the distance from the center of the metalens to the location of the meta-atom. In this work, the change in phase due to the Si pillar meta-atoms is used to satisfy this phase distribution. Figure 2a,b illustrate the transmission and phase change by the meta-atoms as a function of the diameter of the Si pillar. In this calculation, the height and the unit cell size are fixed at 900 nm and 780 nm, respectively, to obtain optimal efficiency. As the diameter increases, the effective index of the propagation mode in Si rod increases. Since the phase delay is proportional to the effective index and the height of the Si pillar (i.e., propagation length), increasing diameter results in larger phase delay. As shown in Fig. 2b, the suggested Si metasurface achieves full 2*π* phase coverage by varying the diameter from 100 to 500 nm while maintaining high transmission. Two Fabry–Perot like resonances are observed near the diameters of 450 nm and 550 nm, resulting in a sharp transmission drop as shown in Fig. 2a. The height of Si pillar is approximately two times higher than effective wavelength (λ/*n*_eff_). Therefore, the multiple resonances are occurred as a result of interferences. These drop in transmission reduce the focusing efficiency. To avoid this, we used alternative diameters with phase delays corresponding to the phase delays at the points where the transmission drops. Where possible, we also employ a larger diameter instead of a small diameter if the corresponding phase delay is same to reduce the fabrication complexity by decreasing the aspect ratio. For example, the phase delay for a diameter of 100 nm (*φ* = 0) is the same as that for a diameter of 500 nm (*φ* = 2π). Then we use an Si pillar with a diameter of 500 nm.

**Figure 3:**
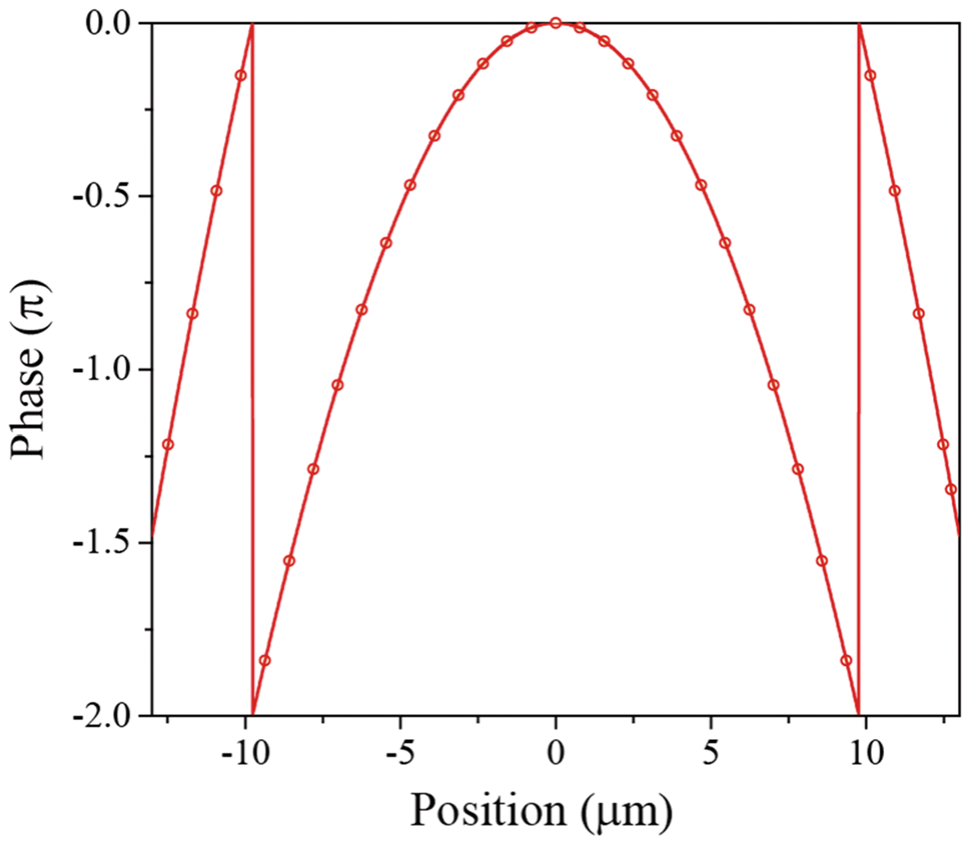
Required (solid line) and realized (dotted line) phase delays for meta-atoms along the radial position of the metalens at a wavelength of 1550 nm. The distance between the dotted lines (unit cell) is 780 nm.

Based on the phase map, we designed the metalens in the core region of the LMA-PCF with a focal length of 30 µm. Note that the area of the entire metalens is not larger than the size of the core. Figure 3 presents the required phase for the focusing of light (solid line) to a focal distance of 30 µm (NA = 0.398) and the phase delay of the designed meta-atoms (dotted line) as a function of their position, where the distance between the dotted lines (meta-atoms) is 780 nm.

### Performance of the metalens

**Figure 4:**
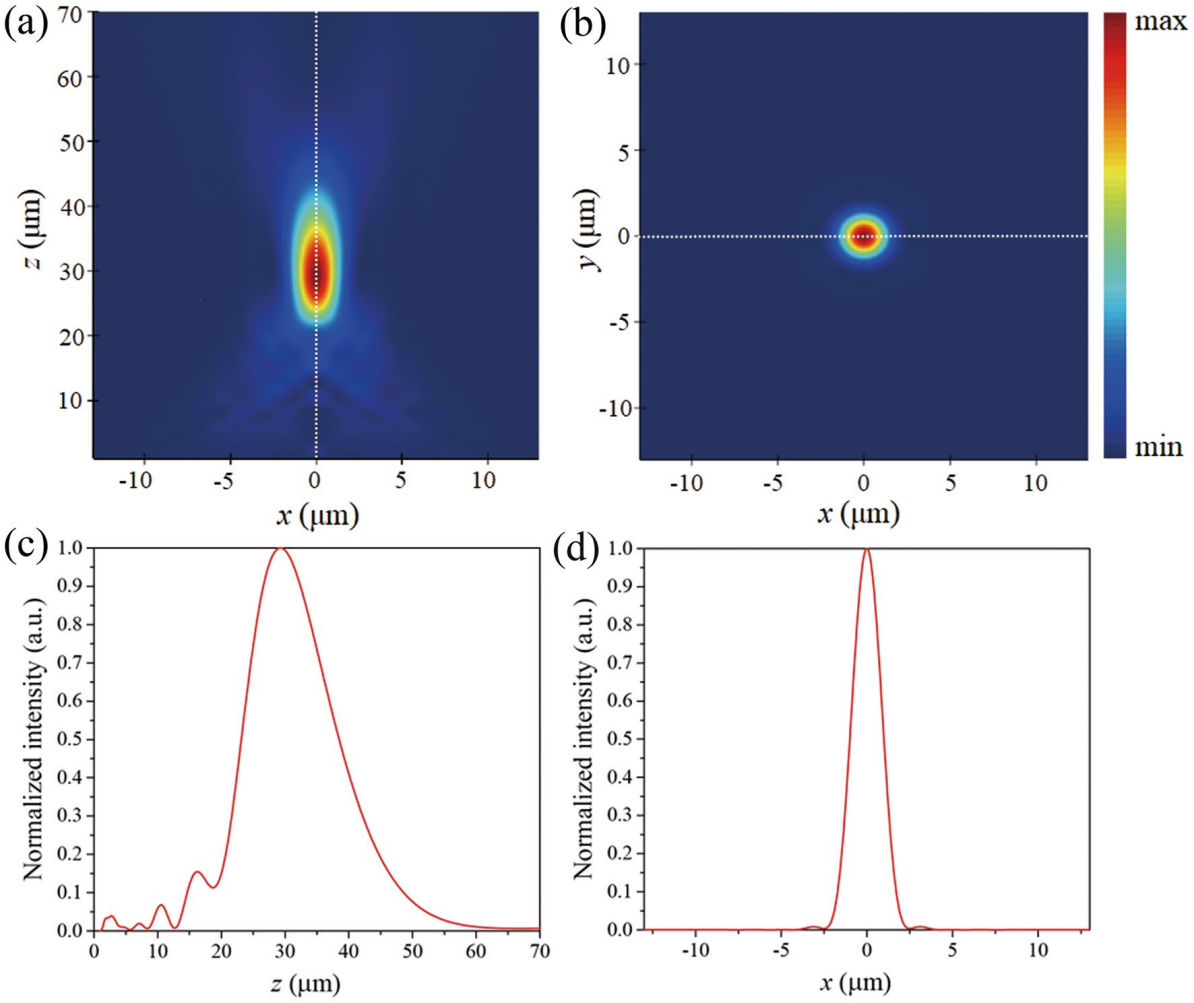
Intensity distributions of the focal spot along the (**a**) cross-section (i.e., *x*–*z* plane) and (**b**) focal plane (x–y plane). (**c**, **d**) Normalized intensity profiles along the white dashed lines in (**a**) and (**b**), respectively.

To verify the focusing effect, we launched the mode source (guided mode) in the LMA-PCF and analyzed the focused light. The focal spot intensity profiles along the cross-section (*x*–*z*) and the focal (*x*–*y*) plane are presented in Fig. 4a,b, respectively. It can be seen that the light intensity is maximized at *x* = 0, *y* = 0, and *z* = 30 µm. To further analyze the optical performance of the metalens, the light intensity distribution along the *z*-direction for *x* = 0 and the *x*-direction at the focal plane are presented in Fig. 4c,d, respectively. The light intensity increases as *z* increases from the position of the metalens to the focal point (*z* = 30 µm), and then it decreases as *z* increases. Therefore, as expected, the light is well focused. As shown in Fig. 4d, the calculated full width at half maximum (FWHM), the beam waist at half-maximum light intensity, is 1.985 µm. We also calculated the efficiency defined as the ratio of the concentrated power inside the focusing region to the total incident power, and a high efficiency of 88% is achieved. Note that this value is approximately 5 times higher than that of a metal-based metalens.

**Figure 5:**
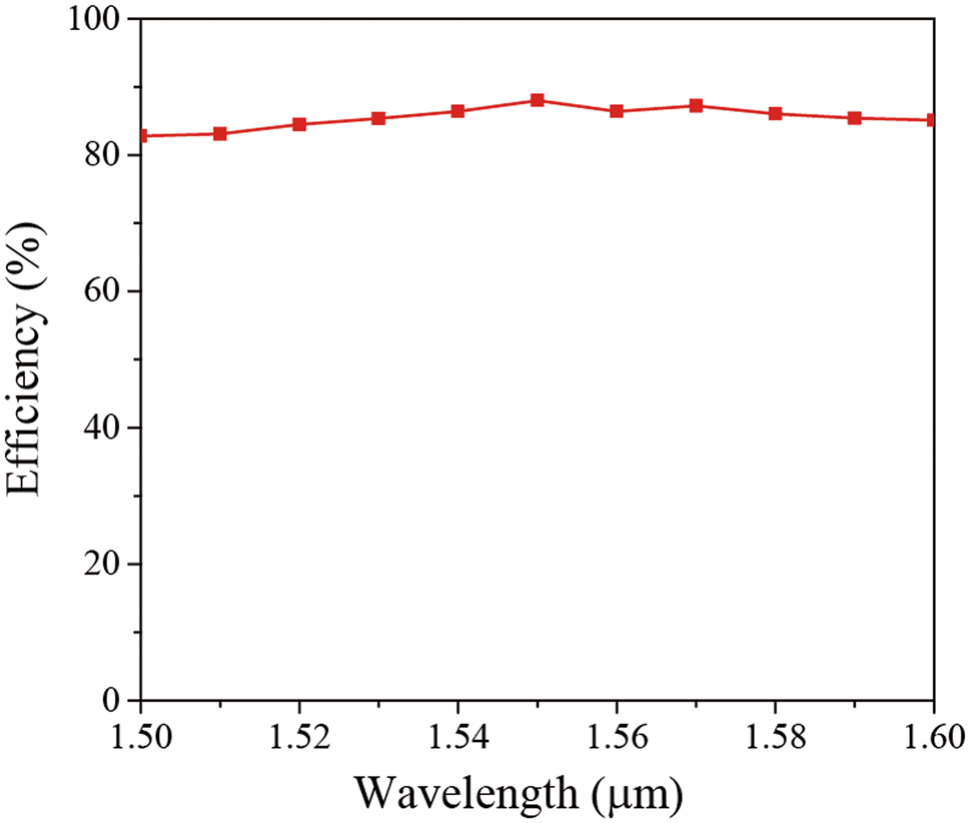
Efficiency of the PCF metalens as a function of wavelength.

We investigated the efficiency variations for the operating wavelength variations from 1.5 µm to 1.6 µm. The same fiber with metalens that has the same parameters as previous calculation was used for the analysis. As shown in Fig. 5, the efficiency is maximized at λ = 1.55 µm, and it is reduced for the others because the designed structure is optimized for the wavelength of 1.55 µm. Since a required phase profile is varied with an operating wavelength, an optimal design varies with operating wavelength, resulting in the efficiency reduction for the other wavelengths. However, the minimum efficiency shows the still high performance of 82.8%. We also calculated the efficiency with a broadband (multi-wavelength) source from λ = 1.5 µm to 1.6 µm. The calculated efficiency maintains a high performance of 83.84%. It should be noted that in high-speed optical fiber telecommunication, the group velocity dispersion affects the system performances. In this case, however, the focal length is just 30 µm, which is very short length considering a dispersion value for conventional PCFs. Therefore, we believe that the performance distortion by the group velocity dispersion is negligibly small.

Fabrication feasibility is an important issue for the practical implementation of an in-fiber metalens. We anticipate that the proposed structure composed of patterned Si pillars can be realized by focused ion beam milling or e-beam lithography with special care taken to align the pillars with the center of the fiber.

## Conclusions

In this paper, we have proposed an in-fiber dielectric metalens for the high-performance focusing of light. A metasurface composed of aperiodic Si pillars provides the desired phase delays with high transmission, which enables the proposed metalens to achieve high efficiency. We have designed the optimal metalens with a focal length of 30 µm at an operating wavelength of 1550 nm. The FWHM and efficiency are 1.985 µm and 88%, respectively, illustrating the superior performances of the proposed metalens compared to a metal-based metalens. We also have investigated the performance of metalens for the various operating wavelength from 1.5 to 1.6 µm. Even though the efficiency is slightly reduced, it still provides a high performance of 82.8%. We believe this integrated in-fiber metalens scheme may lead to a new class of in-fiber optical applications.

## Methods

The metalens simulations were carried out using commercial FDTD software (Lumerical FDTD). We first calculated the transmission and phase delay in the periodic Si pillar structure using a periodic boundary condition. To verify the focusing effect, we calculated the guided mode in the LMA-PCF, and then launched that mode in the metalens structure to analyze the focusing effect. Far-field analysis was conducted to calculate the intensity profile and efficiency.
